# Changes in health-related quality of life in adolescents and the impact of gender and selected variables: a two-year longitudinal study

**DOI:** 10.1186/s12955-022-02035-4

**Published:** 2022-08-18

**Authors:** Hilde Timenes Mikkelsen, Milada Cvancarova Småstuen, Kristin Haraldstad, Sølvi Helseth, Siv Skarstein, Gudrun Rohde

**Affiliations:** 1 Department of Health and Nursing, Faculty of Health and Sport Sciences, University of Agder, Postbox 422, 4604 Kristiansand, Norway; 2 Department of Nursing and Health Promotion, Faculty of Health Sciences, Oslo Metropolitan University, Oslo, Norway; 3 Department of Clinical Research, Sorlandet Hospital, Kristiansand, Norway

**Keywords:** Health-related quality of life, Longitudinal, Adolescent, Resilience, Stress, Loneliness

## Abstract

**Background:**

Increased knowledge about factors that can impact changes in adolescents’ health-related quality of life (HRQOL) is needed. The present study aimed to investigate possible HRQOL changes in adolescents at 14 and 16 years, and assess the impact of sociodemographic factors, gender, pain, self-esteem, self-efficacy, loneliness, and stress on HRQOL changes over time. Further, to assess HRQOL stratified by gender.

**Methods:**

A longitudinal study involving 211 adolescents was conducted. Sociodemographic variables, pain, self-esteem, self-efficacy, loneliness, and stress were all assessed with well-validated instruments. KIDSCREEN-27 was used to measure HRQOL. Data were analyzed using independent t-tests, paired samples t-tests, and linear mixed models for repeated measures.

**Results:**

When all variables were added to the linear mixed models, stress, loneliness, and pain were significantly, independently associated with a reduction in HRQOL change scores for four of the five KIDSCREEN subscales. Time was significantly associated with a reduction in physical and psychological well-being. Self-efficacy and self-esteem were significantly associated with an increase in HRQOL change scores for four and two subscales, respectively. Male gender was significantly negatively associated with changes in social support and peers compared to female gender.

**Conclusion:**

Our results demonstrated a significant decline in adolescents’ HRQOL regarding physical and psychological well-being for the age range 14–16 years. Furthermore, we found that stress, loneliness, and pain have a significant negative impact on HRQOL changes, whereas self-esteem and self-efficacy have a significant positive impact. Our results highlight the importance of increased understanding regarding factors associated with changes in adolescents’ HRQOL to enable accurate and strategic interventions.

**Supplementary Information:**

The online version contains supplementary material available at 10.1186/s12955-022-02035-4.

## Background

Adolescence is an important transitional phase in life, central in the development of capabilities related to health and well-being and where future patterns of adult health are established [1, 2]. According to the World Health Organization, “Investments in adolescent health bring a triple dividend of benefits for adolescents now, for their future adult lives, and for the next generation. Their health and well-being are engines of change in the drive to create healthier, more sustainable societies” [3, p. iv]. To invest in adolescent health, more information about their own perspectives is needed. Thus, in recent years, there has been an increased focus on understanding, improving, and mapping adolescents’ health-related quality of life (HRQOL) [3–5]. HRQOL is a multidimensional construct that includes the individual’s subjective perspectives of the physical, psychological, functional, and social aspects of health [6].

HRQOL is influenced by both individual and environmental characteristics [7]. The adolescence phase is characterized by rapid physical, cognitive, emotional, pubertal, and social changes and an increase in autonomy and independence from caregivers that may lead to vulnerability related to health and HRQOL [1–3, 8]. Previous studies have found that adolescents’ HRQOL seems to deteriorate with age and that girls tend to report lower HRQOL than boys [4, 9, 10]. Self-efficacy, self-esteem, and social support have been found to be positively associated with HRQOL, while low socioeconomic status (SES), pain, loneliness, and stress have been negatively associated with HRQOL in general adolescent populations [11–20]. Most Norwegian adolescents report good health [21]; however, health challenges such as loneliness, stress, and pain seem to be increasing in both Norwegian and international adolescent populations [2, 21–24]. Recent studies have shown that the COVID‐19 pandemic and its protective strategies (e.g., social distancing) have affected the well-being and behavior of adolescents—leading to, for example, increased stress and loneliness, lower life satisfaction, and reduced HRQOL [24–30].

Longitudinal studies of adolescents’ HRQOL can provide a clearer picture of the magnitude and direction of change in adolescents’ HRQOL, help identify factors associated with change over time, and confirm or disconfirm the results of cross-sectional studies [10, 31]. Relatively few studies have investigated how HRQOL changes over time in general adolescent populations. It should be noted that most previous studies are cross-sectional, and most longitudinal studies have focused on specific groups of adolescent populations, such as clinical populations (e.g., selected patient groups). Furthermore, most longitudinal studies have considered only a limited set of potential predictive factors of adolescents’ HRQOL—for example, gender, SES, and age. Thus, there is a need for longitudinal studies that investigate HRQOL in general adolescent populations and include a wide range of potential predictive factors of HRQOL changes. Considering that adolescence is a period when different behaviors are adopted and may track into adulthood, increased knowledge of factors related to changes in adolescents’ HRQOL is necessary to plan effective policies and health promotion interventions [32]. We have previously demonstrated that HRQOL is associated with sociodemographic factors, gender, pain, self-esteem, self-efficacy, loneliness, and stress in a school-based population of 14-to-15-year-old adolescents [14, 20]. In the present study, we aim to further investigate the impact of these specific factors on HRQOL changes over time.

In Norway, the transition from lower secondary to upper secondary school normally involves a change in school institutions for 16-year-old adolescents. School transitions might disrupt established peer groups but also provide opportunities for developing new friendships [33], which may affect adolescents’ well-being [34]. Peer interaction is especially important during adolescence [35], and spending time with friends is considered essential for adolescents’ quality of life [36]. Therefore, longitudinal HRQOL studies covering the transition period from lower secondary to upper secondary school are important.

The primary aims of this study are to investigate possible HRQOL changes in adolescents at 14 and 16 years, and assess the impact of sociodemographic factors, gender, pain, self-esteem, self-efficacy, loneliness, and stress on HRQOL changes over time. Secondary aim was to assess HRQOL stratified by gender.

## Methods

### Sample and data collection

This longitudinal study was part of the “Start Young—Quality of Life and Pain in Generations” study [14], which is a Norwegian mixed-method four-year prospective study in adolescents and their parents. The present study used data collected at baseline (time 1), when the adolescents were 14–15 years (November 2018 to April 2019) [14], and data collected from January to February 2021 (time 2), when the adolescents were 16–17 years. All adolescents that participated at time 1 (N = 696) were sent a text message at time 2 with link to the survey. They received up to three reminders if they did not complete the survey. In total, 211 adolescents (response rate: 30.3%) completed the survey at time 2 and were included in this study.

The data collection was done through a web-based questionnaire. At time 1, the questionnaire was administered and completed in classrooms during school hours. At time 2, the adolescents completed the questionnaire in their spare time. Participants gave their informed consent at the beginning of the survey. We used a safe data server to store the collected data [37]. The questionnaires from time 1 was linked to the questionnaires at time 2 through a mutual ID number. All study procedures were approved by the Norwegian Centre for Research Data (Ref: 60981).

### Measures

An electronic survey tool that consecutively administered the following questionnaires was used. All questionnaires had previously been translated into Norwegian. Most questions included a neutral option, resulting in all items being answered. All questionnaires that used sum scales showed satisfactory Cronbach’s alpha values above .7 (Additional file 1).

The first part of the questionnaire included self-reported data on demographic variables such as gender, age, parental members of the household, parents’ birthplace, and parents’ work status.

*HRQOL* was assessed using the KIDSCREEN-27 questionnaire [38, 39], which is a multidimensional measure of generic HRQOL in adolescents. KIDSCREEN-27 consists of 27 questions organized into five subscales: (1) physical well-being, (2) psychological well-being, (3) autonomy and parent relations, (4) social support and peers, and (5) school environment [38, 40, 41]. The questionnaire is scored on a 5-point Likert scale referring to the last week, indicating either the frequency of certain feelings or behaviors or the intensity of an attitude. Higher scores indicate better HRQOL. In line with the KIDSCREEN handbook [40], Rasch scores were computed for each subscale and transformed into t-values normed to a mean (SD) of 50 (10) which can be compared with international t-values. The Norwegian KIDSCREEN-27 version has been shown to be valid and reliable [39].

*Self-esteem* was assessed using the Rosenberg Self-Esteem scale (RSES) four-item version [42], where respondents rate four self-perception statements on a 4-point Likert scale. The respondent’s scores were summed and divided by 4 to obtain an RSES score ranging from 1 to 4. Higher scores indicate higher levels of self-esteem. The Norwegian RSES four-item version has demonstrated a high degree of correlation (0.95) with the original 10-item version [43] and has previously been used among adolescents [14, 44].

*Self-efficacy* was assessed using the Generalized Self‐Efficacy Scale (GSE), which consists of 10 items that measure optimistic self-beliefs in coping with the challenges, demands, and tasks of life in general [45, 46]. The items are rated on a 4-point scale, and scores on each item are summed and divided by 10 to obtain a GSE score ranging from 1 to 4. Higher scores indicate higher levels of generalized self‐efficacy. The Norwegian GSE has been shown to be valid and reliable [14, 47].

*Loneliness* was assessed using the revised UCLA Loneliness Scale eight-item version (ULS-8), which is rated on a 4-point scale [48]. The total score ranges from 8 to 32. Higher scores indicate a higher degree of loneliness. ULS-8 is an adequate and reliable measure of loneliness among adolescents [48–50], and the Norwegian ULS-8 has shown satisfactory internal consistency [14].

*Stress* was assessed using the Perceived Stress Questionnaire (PSQ), which consists of 30 items that refer to the previous four weeks [51, 52]. The items are rated on a 4-point scale. The PSQ total score is linearly transformed between 0 and 1; PSQ = (raw value – 30) / 90. Higher scores indicate higher levels of perceived stress. Commonly used cutoff levels of stress within PSQ are as follows: low (< .33), medium (.33–.45), moderate (.45–.60), and severe (> .60) [51]. The Norwegian PSQ has demonstrated good reliability and validity [53].

*Pain* was assessed using one question from the Brief Pain Inventory (BPI) [54, 55]—which measures the subjective intensity of pain on average and is rated on a 0–10-point scale, where higher scores indicate more pain. The Norwegian BPI has satisfactory psychometric properties [55] and has been used among adolescents [56]. We also used two questions from the Lübeck Pain-Screening Questionnaire to assess pain duration and pain frequency [57]. These questions were only administered to those who rated 1 or more on the BPI’s “pain on average” question (indicating they had pain). The Lübeck Pain-Screening Questionnaire has shown satisfactory validity, and the Norwegian version has been used among adolescents [58].

### Data analyses

All statistical analyses were conducted using IBM SPSS Statistics (version 27). First, we calculated descriptive characteristics for gender, sociodemographic factors, pain,

self-esteem, self-efficacy, loneliness, and stress at time 1. Then, we used paired sample t-tests to analyze unadjusted differences in HRQOL between time 1 and time 2. Continuous data are presented as means with SDs or medians with min/max and as counts and percentages for categorical variables, as appropriate. Next, we used independent t-tests to analyze unadjusted differences in HRQOL between genders. Gender differences in HRQOL are presented as the estimated means with 95% confidence intervals (CIs).

Finally, we used linear mixed models for repeated measures to assess the impact of gender, sociodemographic factors, pain, self-esteem, self-efficacy, loneliness, and stress on changes in HRQOL over time and time 1 and time 2.The models were fitted separately for each of the five KIDSCREEN-27 subscales as the dependent variables. Time, gender, parental members of the household, parents’ birthplace, parents’ work status, pain on average, self-esteem, self-efficacy, loneliness, and stress were entered into each of the models as fixed effects. These independent variables were collected at time 1 (baseline). We used an unstructured covariance structure with no specific parametric form. The random effects of variables schools (N = 22) and county (N = 4) were also estimated; however, this did not affect the estimates of fixed effects and the overall performance of the models. Thus, we removed the random effects from the models to save statistical power. The results are presented as regression coefficients B with 95% CI and p-values. All analyses were considered exploratory, and no correction for multiple testing was done. Hence, our results should be confirmed by other longitudinal studies. *p* values ≤ .05 were considered statistically significant.

## Results

### Characteristics of the sample

In total, 211 adolescents participated in this longitudinal study, and most were girls (68%). Table 1 shows the descriptive characteristics for gender, sociodemographic factors, pain, self-esteem, self-efficacy, loneliness, and stress at time 1, assessed when the adolescents were 14–15 years. More than two-thirds of the participants lived with both parents, had parents who were both born in Norway, and had parents who were both employed. Among the 161 adolescents (76.3%) who rated one or higher on pain on average (indicating they had pain), about one-third reported they experienced pain often, and 42.2% reported a pain duration of more than three months. The adolescents’ mean (SD) scores for self-esteem and self-efficacy were 3.1 (0.7) and 3.1 (0.4), respectively. The median (min, max) loneliness score was 13 (8, 31), and the mean (SD) stress score was 0.30 (0.16). Details are provided in Table 1.

**Table 1:** Descriptive characteristics for gender, sociodemographic factors, pain, self-esteem, self-efficacy, loneliness, and stress at time 1 (N = 211)

Variables	Time 1 (14–15 years)
Gender, N (%)	
Girls	144 (68.2)
Boys	67 (31.8)
Parental members of the household, N (%)^a^	
Both parents	159 (75.4)
Alternates between two parents	26 (12.3)
One parent and/or other caregivers	26 (12.3)
Parents’ birthplace, N (%)^b^	
Both parents born in Norway	161 (76.3)
One or both parents born in another country	50 (23.7)
Parents’ work status, N (%)^c^	
Both parents employed	168 (79.6)
One parent employed	43 (20.4)
Pain	
Pain on average, median (min, max)^d^	2.0 (0.0, 9.0)
Pain frequency, N (%)^e,f^	
Seldom	68 (42.2)
Sometimes	32 (19.9)
Often	61 (37.9)
Pain duration, N (%)^e,g^	
Pain ≤ 3 months	93 (57.8)
Pain > 3 months	68 (42.2)
Self-esteem, mean (SD)^h^	3.1 (0.7)
Self-efficacy, mean (SD)^i^	3.1 (0.4)
Loneliness, median (min, max)^j^	13 (8, 31)
Stress, mean (SD)^k^	0.30 (0.16)

*SD* Standard deviation

^a^The variable was recoded into three categories: “Both parents,” “Alternates between two parents,” and “One parent and/or other caregivers” (one parent and one step-parent, one parent, other caregivers)

^b^The variable was dichotomized as “Both parents born in Norway” or “One or both parents born in another country” (one parent born in another country, both parents born in another country)

^c^The variable was dichotomized as “Both parents are working” or “One parent is working” (one parent is working, no parents are working)

^d^Range: 0–10, where 10 indicates pain as bad as you can imagine

^e^N = 161

^f^The variable was recoded into three categories: “seldom” (< once/month, once/month), “sometimes” (2–3 times/month, once/week), and “often” (several times/week, every day)

^g^The variable was dichotomized as “Pain ≤ 3 months” (only once, < 1 month, 1–3 months) or “Pain > 3 months” (> 3 months, > 6 months, > 12 months)

^h^Range 1–4, where higher values indicate higher levels of self-esteem

^i^Range 1–4, where higher values indicate higher levels of self-efficacy

^j^Range 8–32, where higher values indicate higher levels of loneliness

^k^Range 0–1, where higher values indicate higher levels of stress

Table 2 shows the descriptive characteristics for HRQOL at time 1 (age: 14–15 years) and time 2 (age: 16–17 years). At time 1, the highest mean (SD) HRQOL score was 53.4 (8.4) for autonomy and parent relations. The lowest HRQOL scores were reported for psychological well-being (46.1 [8.6]). At time 2, the adolescents reported statistically significantly lower HRQOL scores for physical well-being (43.9 [9.5]), psychological well-being (42.7 [8.1]), and school environment (46.4 [9.7]; Table 2) compared to their scores at time 1. Table 3 shows the descriptive characteristics for HRQOL at time 1 and time 2 by gender. At time 1, girls reported statistically significantly lower levels of HRQOL for physical well-being, psychological well-being, and school environment compared to boys. At time 2, girls reported statistically significantly lower levels of HRQOL for psychological well-being, autonomy and parent relations, and school environment (Table 3).

**Table 2:** Descriptive characteristics for health-related quality of life at time 1 and time 2 (N = 211)

	Time 1 (14–15 years)	Time 2 (16–17 years)	*p* values
*HRQOL*			
Physical well-being, mean (SD)^a^	47.0 (9.7)	43.9 (9.5)	** < .001**
Psychological well-being, mean (SD)^a^	46.1 (8.6)	42.7 (8.1)	** < .001**
Autonomy and parent relations, mean (SD)^a^	53.4 (8.4)	52.2 (8.6)	.052
Social support and peers, mean (SD)^a^	48.2 (8.0)	46.9 (9.2)	.086
School environment, mean (SD)^a^	49.4 (8.9)	46.4 (9.7)	** < .001**

Paired-sample t-tests were used to compare differences in HRQOL between time 1 and time 2

*HRQOL* Health-related quality of life, *SD* Standard deviation

*p* values marked in bold indicate *p* ≤ .05

^a^KIDSCREEN subscales. Higher values indicate higher levels of HRQOL

**Table 3:** Descriptive characteristics for health-related quality of life at time 1 and time 2 for girls (N = 144) and boys (N = 67)

	Time 1 (14–15 years)		Time 2 (16–17 years)	
	Girls	Boys	Girls	Boys
Physical well-being, mean [95% CI]^a^	45.6 [44.2–47.1]^b^	49.9 [47.2–52.6]^b^	43.1 [41.6–44.5]	45.9 [43.2–48.6]
Psychological well-being, mean [95% CI]^a^	44.4 [43.1–45.6]^b^	49.7 [47.4–52.0]^b^	41.4 [40.2–42.6]^b^	45.6 [43.4–47.8]^b^
Autonomy and parent relations, mean [95% CI]^a^	52.6 [51.3–53.9]	55.0 [52.8–57.2]	51.0 [49.9–52.1]^b^	55.0 [52.1–57.8]^b^
Social support and peers, mean [95% CI]^a^	48.1 [46.8–49.4]	48.3 [46.4–50.2]	46.4 [44.9–47.9]	48.1 [45.9–50.3]
School environment, mean [95% CI]^a^	48.3 [47.0–49.6]^b^	51.6 [49.1–54.1]^b^	45.2 [43.7–46.8]^b^	49.1 [46.7–51.6]^b^

Continuous variables analyzed using independent t-tests. HRQOL, health-related quality of life; CI, confidence interval

^a^KIDSCREEN subscales. Higher values indicate higher levels of HRQOL

^b^Significant difference between genders, p ≤ 0.05

Table 4 shows the adjusted associations between time, gender, sociodemographic factors, pain, self-esteem, self-efficacy, loneliness, stress, and changes in HRQOL. When all variables were added into the models, stress, loneliness, and pain were all significantly, independently, and negatively associated with a reduction in HRQOL for four of the five KIDSCREEN subscales. Stress had its highest negative effect on autonomy and parent relations (B =  − 2.00; 95% CI [− 2.61 to − 1.39]), loneliness had its highest negative effect on social support and peers (B =  − 0.95; 95% CI [− 1.13 to − 0.77]), and pain had its highest negative effect on school environment (B =  − 0.68; 95% CI [− 1.07 to − 0.29]). Time was significantly associated with a reduction in physical well-being (B =  − 1.50; 95% CI [− 2.76 to − 0.26]) and psychological well-being (B =  − 1.22; 95% CI [− 2.11 to − 0.33]). In contrast, self-efficacy was significantly positively associated with an increase in HRQOL considering four of the five KIDSCREEN subscales, with the highest positive effect on school environment (B = 5.73; 95% CI [3.72 to 7.74]). Furthermore, self-esteem was significantly associated with an increase in physical well-being (B = 1.63; 95% CI [0.08 to 3.16]) and psychological well-being (B = 3.31; 95% CI [2.28 to 4.36]). Gender was only significantly associated with changes in social support and peers. For this subscale, being a boy was associated with lower HRQOL (B =  − 1.76; 95% CI [− 3.42 to − 0.11]) compared to being a girl. The selected sociodemographic variables were not significantly associated with changes in HRQOL—except for parents’ work status, which indicated that when both parents were employed (B = 2.41; 95% CI [0.21 to 4.62]), this was significantly associated with an increase in the adolescents’ physical well-being compared to when only one parent was employed. Details are provided in Table 4.

**Table 4:** Adjusted associations between time, gender, sociodemographic factors, pain, self-esteem, self-efficacy, loneliness, stress and changes in health-related quality of life estimated with linear mixed model analyses (N = 211)

	Physical well-being			Psychological well-being		
	B	95% CI	*p* value	B	95% CI	*p* value
Time						
2021	− **1.50**	− **2.76 to** − **0.26**	**.018**	− **1.22**	− **2.11 to** − **0.33**	**.007**
2019 (Ref.)	1			1		
Gender						
Boy	− 0.44	− 2.38 to 1.51	.659	0.70	− 0.54 to 1.94	.268
Girl (Ref.)	1			1		
Parental members of the household						
Both parents	0.19	− 2.38 to 2.75	.886	− 0.71	− 2.40 to 0.97	.406
Alternates between two parents	0.28	− 2.68 to 3.24	.853	− 0.51	− 2.50 to 1.47	.612
One parent and/or other caregivers (Ref.)	1			1		
Parents’ birthplace						
Both parents born in Norway	0.45	− 1.65 to 2.54	.675	− 0.14	− 1.48 to 1.19	.834
One or both parents born in another country (Ref.)	1			1		
Parents’ work status						
Both parents are working	**2.41**	**0.21 to 4.62**	**.032**	1.14	− 0.03 to 2.58	.121
One parent is working (Ref.)	1			1		
Pain on average	− **0.49**	− **0.90 to** − **0.09**	**.017**	− **0.54**	− **0.81 to** − **0.27**	** < .001**
Self-esteem	**1.63**	**0.08 to 3.16**	**.038**	**3.31**	**2.28 to 4.36**	** < .001**
Self-efficacy	**4.80**	**2.68 to 6.92**	** < .001**	**2.31**	**0.90 to 3.73**	**.001**
Loneliness	− **0.23**	− **0.43 to** − **0.04**	**.017**	− **0.49**	− **0.62 to** − − **0.36**	** < 0.001**
Stress	− **1.10**	− **1.76 to** − **.45**	**0.001**	− **1.17**	− **1.61 –** − **.73**	** < 0.001**

	Autonomy and parent relations			Social support and peers		
	B	95% CI	*p* value	B	95% CI	*p* value
Time						
2021	0.67	− 0.53 to 1.86	.272	0.70	− 0.56 to 1.96	.275
2019 (Ref.)	1			1		
Gender						
Boy	0.35	− 1.41 to 2.11	.695	− **1.76**	− **3.42 to** − **0.11**	**.037**
Girl (Ref.)	1			1		
Parental members of the household						
Both parents	0.77	− 1.57 to 3.13	.518	0.44	− 1.83 to 2.73	.701
Alternates between two parents	− 0.11	− 2.86 to 2.64	.936	1.01	− 1.71 to 3.74	.465
One parent and/or other caregivers (Ref.)	1			1		
Parents’ birthplace						
Both parents born in Norway	1.82	− 0.07 to 3.72	.059	− 0.06	− 1.85 to 1.72	.947
One or both parents born in another country (Ref.)	1			1		
Parents’ work status						
Both parents are working	1.61	− 0.42 to 3.63	.119	− 1.15	− 3.1 to 0.80	.245
One parent is working (Ref.)	1			1		
Pain on average	− **0.45**	− **0.82 to** − **0.07**	**.020**	− 0.23	− 0.60 to 0.14	.224
Self-esteem	− 0.07	− 1.51 to 1.36	.917	0.11	− 1.31 to 1.5	.876
Self-efficacy	0.79	− 1.17 to 2.76	.427	**1.94**	**0.01 to 3.87**	**.049**
Loneliness	− 0.09	− 0.27 to 0.09	.323	− **0.95**	− **1.13 to** − **0.77**	** < .001**
Stress	− **2.00**	− **2.61 to** − **1.39**	** < .001**	− 0.37	98 – .23	.228

	School environment		
	B	95% CI	*p* value
Time			
2021	− 1.27	− 2.61 to 0.06	.062
2019 (Ref.)	1		
Gender			
Boy	− 0.58	− 2.28 to 1.12	.503
Girl (Ref.)	1		
Parental members of the household			
Both parents	− 1.94	− 4.29 to 0.42	.106
Alternates between two parents	− 1.19	− 4.02 to 1.64	.409
One parent and/or other caregivers (Ref.)	1		
Parents’ birthplace			
Both parents born in Norway	− 0.03	− 0.87 to 1.80	.972
One or both parents born in another country (Ref.)	1		
Parents’ work status			
Both parents are working	1.21	− 0.80 to 3.22	.238
One parent is working (Ref.)	1		
Pain on average	− **0.68**	− **1.07 to** − **0.29**	**.001**
Self-esteem	1.10	− 0.38 to 2.58	.146
Self-efficacy	**5.73**	**3.72 to 7.74**	** < .001**
Loneliness	− **0.20**	− **0.39 to** − **0.02**	**.030**
Stress	− **1.33**	− **1.9 to** − **0.70**	** < .001**

Linear mixed model analyses were performed separately for each of the five KIDSCREEN-27 subscales as the dependent variables

HRQOL was analyzed with KIDSCREEN-27 subscales. Higher values indicate higher levels of HRQOL

B, Unstandardized coefficient; CI, Confidence interval; HRQOL, Health-related quality of life

*p* values marked in bold indicate *p* ≤ .05

## Discussion

This longitudinal study aimed to investigate possible HRQOL changes in adolescents at 14 and 16 years, and assess the impact of sociodemographic factors, gender, pain, self-esteem, self-efficacy, loneliness, and stress on HRQOL changes over time. Further, we aimed to assess HRQOL stratified by gender. Our results showed that stress, loneliness, and pain had a significantly negative impact on HRQOL changes, whereas self-esteem and self-efficacy had a significantly positive impact. Time was significantly associated with a reduction in physical and psychological well-being and male gender was significantly negatively associated with changes in social support and peers compared to female gender. Girls reported statistically significantly lower levels of HRQOL for three of the KIDSCREEN subscales at time 1 and at time 2 compared to boys.

Our results showed that HRQOL decreased with age; however, this result was only significant for the physical well-being and psychological well-being scales. It is important to note that while the adolescents’ HRQOL scores reported at time 1 are comparable to European norms, their HRQOL scores reported at time 2 are notably lower compared to European norms [40]. This should be viewed in light of the COVID-19 pandemic. During the pandemic, several studies have reported lower HRQOL scores in adolescents compared to the results of previous studies in adolescent populations [26–30].

Stress at age 14–15 years was significantly negatively associated with a reduction in HRQOL change scores in four KIDSCREEN subscales, although the stress score in our sample indicated low levels of stress. In line with previous findings [14], we found that stress had the highest negative effect on the KIDSCREEN subscale autonomy and parent relations, underscoring the need to be aware of the negative impact stress seems to have on this HRQOL dimension, which reflects the quality of adolescent and parent interactions, the feeling of love and support by family, and adolescents’ perceived autonomy [40]. Our findings confirm that stress is a considerable risk factor for adolescents’ HRQOL [14, 17], and add to existing knowledge by indicating that this is evident even with low levels of stress.

Higher levels of loneliness were associated with a decrease in HRQOL change scores, confirming the result from a previous cross-sectional study [14]. Adolescence is a life phase where biological, cognitive, social, and demographic changes may influence loneliness [59]. Furthermore, feelings of loneliness may have increased during the COVID-19 pandemic [24]. Hence, we emphasize that loneliness should be viewed as a significant threat to changes in adolescents’ HRQOL during and after the pandemic.

The median intensity of pain reported at time 1 of 2.0 is not considered high. Nevertheless, pain was significantly associated with a reduction in HRQOL change scores in four KIDSCREEN subscales. Thus, our results support previous studies demonstrating a negative association between pain and HRQOL in adolescents [14, 20] and indicate that this is evident even with low levels of pain. Moreover, we found that pain had its highest negative effect on school environment, which explores the adolescents’ feelings about school, the perception of their cognitive capacity, concentration, and learning; and their views of the relationship with their teachers [40]. Hence, we accentuate the need for interventions aiming to reduce the negative impact pain seems to have on changes in HRQOL related to the school environment.

Our results confirm the positive association between higher levels of self-esteem and self-efficacy and an increase in HRQOL change scores. Self-efficacy and self-esteem are both considered resilient factors [60]. Resilience refers to having a relative resistance to risk experiences or overcoming adversity or stress [61]. Thus, our results emphasize the importance of resilience factors for HRQOL over time and call attention to the need for interventions aimed at increasing adolescents’ resilience. Resilience interventions can increase adolescents’ protective behaviors and coping skills—which can help them manage daily stressors, allowing for greater well-being and academic success [62]. Moreover, resilience factors may protect adolescents’ mental health in times of crisis, such as the COVID-19 pandemic [63].

Girls reported lower levels of HRQOL compared to boys at age 14–15 years and at age 16–17 years. This confirms findings from previous longitudinal studies among adolescents [4, 9, 10]. Nevertheless, in four KIDSCREEN subscales, we found no significant association between gender and changes in HRQOL. For these subscales, our results may indicate that gender-related differences in HRQOL remained unchanged during youth. A possible explanation may be that gender is an important factor concerning HRQOL but that part of the gender-related differences in HRQOL might be explained by gender-related differences within other factors associated with HRQOL [10, 14, 15]. Surprisingly, for the subscale social support and peers, we found that male gender was associated with lower HRQOL scores compared to female gender. The subscale social support and peers explores adolescents’ perceived support and the quality of the interaction between adolescents and peers [40]. Hence, our findings may be explained by previous research showing that adolescent boys report higher levels of social loneliness, which refers to the absence of a broader accessible and supportive social network, compared to girls [59]. Moreover, loneliness in boys is considered more sensitive to their interpersonal relationships [64].

A negative association between changes in HRQOL and low SES was not supported by our findings—except for the factor parents’ work status, which showed that both parents being employed was associated with higher scores in the adolescents’ physical well-being compared to when only one parent was employed. We have searched similar studies to find an explanation for this but have found none. Thus, we recommend future studies to further explore our findings. A possible explanation for our results regarding SES may be that other factors (e.g., stress, loneliness, and self-efficacy) outweighed the effect of SES. Furthermore, the results may have been influenced by high SES in our sample.

### Strengths and limitations

The main strengths of this study are the longitudinal design and use of a sample that is representative of an unselected adolescent population and the inclusion of a wide range of potential predictive factors associated with a change in HRQOL. All these factors were assessed with well-validated instruments. The overall response rate was only 30.3%, which is a limitation. Attrition can be a major methodological problem in longitudinal studies and may deteriorate the generalizability of findings [65]. The scores for sociodemographic factors, pain, self-esteem, self-efficacy, loneliness, and stress among the responders (N = 211) are similar to previous findings among the potential participants (N = 696) [14], indicating that the responders at time 2 were similar to the non-responders. However, the responders consisted of more girls (68%) compared to the sample of potential participants (57.5%). This may have influenced the results. Furthermore, it is important to note that more than two-thirds of the participants lived with both parents, had parents who were both born in Norway, and had parents both employed, indicating high SES. Thus, the results may not be representative of adolescents from low SES families. This should be considered when interpreting our results. Moreover, we did not control for other possible confounders—for example, depression, anxiety, bullying, and physical activity. Hence, we recommend controlling for other confounders in future studies.

### Implications

Our results provide important insights into HRQOL changes in adolescents during the transition period from lower secondary to upper secondary school, from 14 to 16 years, and the impact of gender, sociodemographic factors, pain, self-esteem, self-efficacy, loneliness, and stress on HRQOL changes over time. The findings provide insight into a complex life phase and confirm that several factors can influence changes in adolescents’ HRQOL, such as stress, loneliness, pain, self-esteem, and self-efficacy. We recommend future health-promoting interventions among adolescents to target these factors. Considering that the ongoing COVID‐19 pandemic is leading to increased stress and loneliness and reduced HRQOL in adolescents [25–30], an increased understanding of factors associated with HRQOL seems highly relevant.

Based on previous research [11, 14, 15, 17] and our results showing the importance of self-esteem and self-efficacy for HRQOL, we recommend an increased focus on resilience-promoting interventions at school. School interventions can support positive growth and changes to all students within a class, although with more significant effects in the at-risk group [62, 66]. Teachers are considered an important resource in the development of resilience, as they are more likely to know the students’ lived experiences and current help-seeking and coping strategies [62]. We also highlight the need to involve parents regarding resilience promotion. The involvement of parents is considered a key component of effective resilience interventions, as parents are important influencers and role models for adolescents [66, 67].

In future studies to explore our findings more thoroughly, the sample should be extended and include more boys and adolescents with an immigrant background, with low SES, and who live with only one parent. Future studies may also analyze the development of HRQOL in adolescents over a longer period and include possible confounders not included in the present study, such as depression, anxiety, bullying, and physical activity. Furthermore, qualitative data are needed to gain more in-depth knowledge of factors associated with changes in adolescents’ HRQOL over time.

## Conclusions

Our study provides important insight into changes in adolescents’ HRQOL at two time points when they were 14 and 16 years, and into factors associated with these changes. We found a significant decline in adolescents’ HRQOL regarding physical and psychological well-being during these two years. Further, we found that stress, loneliness, and pain have a significant negative impact on HRQOL changes, whereas self-esteem and self-efficacy have a significant positive impact. Our results highlight the importance of increased understanding regarding factors associated with changes in adolescents’ HRQOL to be able to intervene accurately and strategically.

## Supplementary Information

**Additional file 1.** Cronbach’s alpha values for instruments used in this study. A table providing cronbach’s alpha values for KIDSCREEN-27, Generalized Self-efficacy scale, Rosenberg Self-Esteem Scale, UCLA Loneliness Scale, Perceived Stress Questionnaire.

## Data Availability

The datasets used and/or analyzed during the current study are not publicly available due to General Data Protection Regulation laws but are available from the corresponding author on reasonable request and with permission from the Norwegian Centre for Research Data.

## Abbreviations

BPI: Brief pain inventory
CI: Confidence interval
GSE: General self-efficacy scale
HRQOL: Health-related quality of life
LPQ: Lübeck Pain-Screening Questionnaire
PSQ: Perceived Stress Questionnaire
RSES: Rosenberg self-esteem scale
SD: Standard deviation
SES: Socioeconomic status
ULS: UCLA loneliness scale
