# Supplementary material for: Changes in health-related quality of life in adolescents and the impact of gender and selected variables: a two-year longitudinal study
Source: Health Qual Life Outcomes. 2022 Aug 18;20:123. doi: 10.1186/s12955-022-02035-4 (PMC9387404; doi:10.1186/s12955-022-02035-4)
Supplement: Supplementary file 1 — Additional file 1. Cronbach’s alpha values for instruments used in this study. A table providing cronbach’s alpha values for KIDSCREEN-27, Generalized Self-efficacy scale, Rosenberg Self-Esteem Scale, UCLA Loneliness Scale, Perceived Stress Questionnaire. [file 12955_2022_2035_MOESM1_ESM.docx]

**Additional file 1: Cronbach’s alpha values for instruments used in this study**

| Factors | Instruments | Number  of items | α^ab^ | α^ac^ |
| --- | --- | --- | --- | --- |
| HRQOL | KIDSCREEN-27 |  |  |  |
|  | Physical well-being | 5 | .81 | .81 |
|  | Psychological well-being | 7 | .88 | .88 |
|  | Autonomy and parent relations | 7 | .77 | .79 |
|  | Social support and peers | 4 | .79 | .78 |
|  | School environment | 4 | .80 | .83 |
| Self-efficacy | Generalized Self‐Efficacy Scale | 10 | .86 |  |
| Self-esteem | Rosenberg Self-Esteem Scale | 4 | .81 |  |
| Loneliness | UCLA Loneliness Scale | 8 | .82 |  |
| Stress | Perceived Stress Questionnaire | 30 | .94 |  |

ª Cronbach’s alpha coefficient values in this study; ^b^ at time 1; ^c^ at time 2

HRQOL, health-related quality of life
